# Qualification of the human Reconstructed Intestine Micronuclei Cytome assay for site-of-contact genotoxic hazard identification

**DOI:** 10.1016/j.namjnl.2025.100015

**Published:** 2025-03-28

**Authors:** Hui Kheng Lim, Christopher Owen Hughes, Timothy Landry, Choon Wee Joseph Tan, Seyoum Ayehunie, Benjamin Paul Chapman Smith

**Affiliations:** a Future Ready Food Safety Hub (a Joint Initiative of A*STAR, SFA and NTU), Nanyang Technological University, Singapore; b Monell Chemical Senses Center, Philadelphia, USA; c MatTek Corporation, Ashland, Massachusetts, USA

**Keywords:** Micronuclei, Ricyt assay, Site-of-first-contact, Genotoxic hazard identification, Epiintestinal™

## Abstract

While valuable for hazard identification, *in vitro* genotoxicity tests that are conducted in 2D monolayer cell cultures possess limitations to accurately envisage *in vivo* outcomes and may result in unnecessary follow-up *in vivo* studies. One limitation is their relevance to actual human exposure and the assessment of genotoxicity in tissues representative of “sites-of-first-contact” with genotoxic agents. The revised OECD testing guidelines had emphasized the need for considering site-of-first-contact effect when carrying out genotoxic hazard investigations. To date, only *in vivo* studies can adequately address the oral/gastrointestinal route of exposure and there exists no validated *in vitro* assay for ingestible substances. Previously, we described the development of a new human Reconstructed Intestine Micronuclei Cytome (RICyt) assay using EpiIntestinal™ microtissues for site-of-contact genotoxic hazard identification of orally ingested materials. The human small intestine is where approximately 90 % of the digestion and adsorption of food occurs and a major exposure site of ingested genotoxicants, thus a key site for evaluation. Here, we have investigated on the qualification of the RICyt assay in genotoxic hazard identification. The assay platform was challenged with a training set of 16 test materials which include standard reference genotoxins, non-genotoxins and food relevant substances with various mode of actions. The overall RICyt accuracy was 93.3 % with sensitivity of 83.3 % and specificity of 100 %. Majority of the test materials were correctly identified though yielded one false negative (KBrO_3_) and one equivocal outcome (EUG). MCT, a known genotoxin, with no known effect on the GI tract, did not induce significant MN, showcasing the ability of the assay to detect tissue specific genotoxicity. These results emphasize that the RICyt assay is promising *in vitro* tool for genotoxic hazard identification of orally ingested substances.

## Highlights

• First *in vitro* assay to address site-of-contact genotoxic hazard identification for oral or GI route of exposure.
• Assay acceptance criteria have been established to determine/ classify genotoxic status of test articles.
• Assay accuracy established for genotoxic risk identification.
• Can potentially reduce the unnecessary usage of animal experimentation once sufficiently validated.

## Introduction

With the recent advent of novel food as alternative source of food ingredients, there is need for more relevant, route specific *in vitro* or *in vivo* safety tests. The European Food Safety Authority (EFSA) recommends a genotoxicity testing battery including a reverse mutation assay, an *in vitro* mammalian cell micronucleus and an *in vivo* genotoxicity assay, usually the micronucleus assay in bone marrow immature erythrocytes for further confirmation. Although these tests provide important information on the potential for genotoxicity, they do not provide site-of-first contact information. To improve the assessment of genotoxic risk in humans, it is important to develop assays in relevant models that closely represent the target tissues exposed. For food-relevant materials, the gastrointestinal (GI) tract represents a more relevant target organ because of the potential higher exposure, especially for substances that are poorly absorbed and scarcely bioavailable to systemic circulation (More et al., 2021). Effort have been made to develop *in vivo* micronucleus test in the GI tract (Okada et al., 2022; Coffing et al., 2011). There is anatomical difference between human and murine GI tract. Innate and immune interactions in the small intestinal of murine and humans are different (Yue et al., 2014). The small intestinal villi of mice are much taller than those in humans to provide larger surface for absorption and to compensate for the lack of plicae, which are found in human small intestine (Nishiyama et al., 2016; Sheahan and Jervis, 1976). The small intestine is about 20-fold longer than mice (700 vs 33 cm) (Hugenholtz and de Vos, 2018). Rodents are viewed as the “gold standard” which are commonly used as a test species in safety assessment, have a poor predictivity rate of 40 %–65 % (Olson et al., 2000; Bailey et al., 2014; Monticello et al., 2017; Clark and Steger-Hartmann, 2018; Atkins et al., 2020; Rooney et al., 2021; Friedman et al., 2023). Though animal models are vitally important in genotoxicity testing, the inherent different between animals and human might not allow a direct comparison and straight draw of conclusion.

The emergence of New Approach Methodologies (NAMs) in the scientific field has gained significant momentum in the past decade. NAMs encompass *in vitro, in chemico* or *in silico* approach that, when used in solitary, or in combination with other methods, improves overall chemical safety assessment. NAMs promotes the establishment of more relevant methodologies via the use of more species-relevant tools to model specific biological or physiological condition to allow better reflect of the *in vivo* complexities and potentially predict human responses. The aim of NAMs is not to directly replace *in vivo* test; rather it provides more relevant information for chemical safety assessment. While NAMs focus on human health hazard and risk assessment, using different approaches, it cannot be utterly represent all aspect of human complexities. Currently, the utilization of NAMs in food safety assessment remains limited (Friedman et al., 2023).

Our team had previously developed Reconstructed Intestine Micronuclei Cytome (RICyt) assay with the specific aim to address site-specific DNA damage assessment and determine mode of cell death (Lim et al., 2022). We proposed the use of 3D reconstructed human epithelial models, EpiIntestinal™, for the RICyt assay protocol. The EpiIntestinal™, possess authentic structural, biochemical, physiological, and mechanistic properties that are different compared to 2D cell models of the gut (Ayehunie et al., 2018). In addition, this *in vitro* model is reconstructed from primary cells derived from ileum of consent human donor (Ayehunie et al., 2014; Markus et al., 2021). Therefore, the data deduced from the *in vitro* 3D tissue model may provide higher level of human relevance than 2D cell models when the route of exposure is either oral or gastrointestinal. Additional advantages include that the 3D EpiIntestinal™ model can be maintained *in vitro* for a longer period of time (>40 days), which allows repeated dosing studies similar to *in vivo* experiments (Peters et al., 2019; Fedi et al., 2021). Currently, the RICyt assay is promising predictive tool in hazard identification of genotoxic materials though at the proof-of-concept stage. The final goal is not to replace the *in vivo* model but to gather additional evidence that can potentially reduce the unnecessary usage of animal experimentation once validated.

Here we report a within-laboratory study to assess the qualification of RICyt assay protocol in predicting the genotoxicity of orally ingested materials. Our aim is to assess assay predictivity using a training set of 16 compounds. To assess assay sensitivity 8 compounds known to be genotoxins *in vivo* and *in vitro* (Table 1) were included in the study. To establish the assay specificity, 8 compounds known to be non-genotoxins either *in vivo* or *in vitro* (Table 2) were tested. The test compounds can be broadly categorized into standard genotoxic reference materials, non-genotoxic materials, conventional and novel food ingredients, food additives, food contaminants and toxic metabolites synthesized by plant or from thermal-processed food. Some of the test compounds were chosen from the recommended chemical list published by Kirland et al. (Kirkland et al., 2016) to evaluate the sensitivity and specificity of new/modified mammalian model genotoxicity tests. The rest of the food-related test compounds were selected from multiple online literature sources and their genotoxicity status were checked against findings published by EFSA, to probe the assay performance in the food safety space.

**Table 1:** List of tested genotoxins, their mode of action and a comparison of expected results between MN in EpiIntestinal™ tissues and *in vitro*/ *in vivo* results.

		Genotoxic profile						
Chemicals	CAS number	Ames test	*In vivo* genotoxicity tests	*In vitro* genotoxicity tests	MoA	Requirement for metabolism	RICyt	Conclusion
N-Ethyl-N-nitrosourea	759-73-9	+	+ CA, MN, comet	+ MN, CA, MLA, HPRT mutations and UDS	Clastogen	No	Positive	Correct
Glycidamide	5694-00-8	-	+ MN & DNA adduct	+ DNA adduct & comet	Clastogen	No	Positive	Correct
Paclitaxel	33069-62-4	-	+ MN in bone marrow and liver	+ MN, polyploidy in a CA test and in MLA	Aneugen	No	Positive	Correct
Aflatoxin B1	1162-65-8	+	+ MN, CA, UDS − comets	+ CA, MN, HPRT mutations at low concentrations +S9	Clastogen	Yes CYP3A4 & CYP1A2	Positive	Correct
Ethyl methanesulfonate	62-50-0	+	+ MN, comet and CA	+ MN, comet, CA, SCE and HPRT mutations	Clastogen	No	Positive	Correct
Monocrotaline	315-22-0	+	+ DNA adduct, DNA crosslinking and MN	+ DNA adduct, DNA crosslinking, MN and SCE	Clastogen	Yes CYP3A4	Negative	Correct
β-asarone	5273-86-9	+	+ UDS - MN	+ MN, comet and SCE	Clastogen (*in vitro*)	Yes CYP3A4	Negative	Correct
Potassium bromate	7758-01-2	+	- MN to bone marrow, stomach + comet to small intestine	+ MN	Clastogen	No	Negative	Incorrect

MoA, mechanism of action; CYP, Cytochrome P450; CA, chromosomal aberrations; MN, micronuclei; HPRT, hypoxanthine-guanine phosphoribosyl transferase; UDS, unscheduled DNA synthesis; SCE, sister chromatid exchange; MLA, mouse lymphoma.

**Table 2:** List of tested non-DNA-reactive chemicals (including non-genotoxins) , their mode of action and a comparison of expected results between MN in EpiIntestinal™ tissues and *in vitro*/ *in vivo* results.

		Genotoxic profile						
Chemicals	CAS number	Ames test	*In vivo* genotoxicity tests	*In vitro* genotoxicity tests	MoA	Requirement for metabolism	RICyt	Conclusion
Eugenol	97-53-0	-	+ MN only at very high doses. Equivocal	+ DNA adduct & CA. Equivocal	-	Yes UGT1A6 & UGT2B1	Equivocal	Equivocal
Curcumin	458-37-7	-	- MN and comet	+ MN and DNA adduct. Equivocal	Clastogen (*in vitro*)	No	Negative	Correct
5-Hydroxymethyl furfural	67-47-0	-	- MN	+ Comet and SCE - for MN	Clastogen (*in vitro*)	Yes SULT	Negative	Correct
Menaquinone 7	2124-57-4	Not well studied	-	-	-	No	Negative	Correct
Melamine	108-78-1	-	-	-	-	No	Negative	Correct
Astaxanthin esters	7542-45-2	-	-	-	-	Yes CYP1A1 & CYP1A2	Negative	Correct
Aspartame	22839-47-0	-	- MN, comet and CA	- CA and MN - SCE	-	No	Negative	Correct
d,l-Menthol	15356-70-4	-	-	-	-	Yes UGT & Oxidation	Negative	Correct

MoA, mechanism of action; CYP, Cytochrome P450; CA, chromosomal aberrations; MN, micronuclei; SCE, sister chromatid exchange; SULT, sulfotransferase; UGT, UDP glucuronosyltransferases.

## Material and methods

### Test chemicals

N-Ethyl-N-nitrosourea (CAS 759–73–9), N3385, Paclitaxel (CAS 33069–62–4), PHL89806, Potassium bromate (CAS 7758–01–2), 309087, Monocrotaline (CAS 315–22–0), C2401, Glycidamide (CAS 5694–00–8), 04704, 5-Hydroxymethylfurfural (CAS 67–47–0), W501808, Melamine (CAS 108–78–1), M2659, Eugenol (CAS 97–53–0), E51791, Astaxanthin esters (CAS 7542–45–2), 1044210, D,L-Menthol (CAS 15356–70–4), CRM40467, Aspartame (CAS 22839–47–0), W700655, Menaquinone 7 (CAS 2124–57–4), PHR2363, Ethyl methanesulfonate (CAS 62–50–0), M0880, Curcumin (CAS 458–37–7), C1386 and β-asarone (CAS 5273–86–9), 02890590 were purchased from Sigma Aldrich (St Louis, MO). Aflatoxin B1 (CAS 1162–65–8), 11293, were purchased from Caymen (Ann Arbor, MI). Test chemical stock solutions were prepared in anhydrous DMSO (Sigma Aldrich, St. Louis, MO) or in water.

### 3D tissue culture

EpiIntestinal™ tissue (SMI-200-FT v2.0) was purchased from MatTek Corporation® (Ashland, MA). Briefly, the microtissues were cultured on Transwell™ plates by seeding primary human intestinal fibroblasts and small intestinal epithelial cells in permeable tissue culture inserts (surface area= 0.6 cm^2^, membrane pore size = 0.4 μm) at 37°C at submerged condition followed by air-liquid interface (ALI).The co-cultures were maintained in SMI-100-MM media (MatTek Corporation®) for a duration of 14 days at 37°C, 5 % CO_2_/95 % relative humidity until the microtissues were fully differentiated and stratified. The mature tissues are sealed in 24-well agar-plugged plates and shipped to our laboratory in Singapore. Upon arrival the tissues were re-equilibrated in fresh SMI-100-MM for overnight recovery prior to the test chemical treatment.

### Experimental design and treatment

The RICyt assay treatment regimen was developed and performed as previously described in Lim et al. (Lim et al., 2022). Prior to test article exposure, the microtissues were rinsed twice with Hanks Balanced Salts Solution containing calcium and magnesium (HBSS +Ca/Mg). Media containing 0.1 % DMSO or 10 % H_2_O were applied to tissues to serve as the vehicle control. Dose selections were based on literature review, cytotoxicity or test material solubility. If the maximum test concentration is based on cytotoxicity, the highest test concentration should aim to achieve 55±5 % cytotoxicity. If test material solubility is limited factor, the highest concentration analysed should produce turbidity or a precipitate visible by eye or with the aid of microscopy (OECD 2023). Test chemicals were diluted in complete culture media. All microtissues were treated apically by adding 120 µl of the test article or vehicle control every other day (from days 0–6) and daily (from days 7–9). At all times, the tissues were fed with SMI-100-MM media basolaterally every other day to support the tissue growth.

### Tissue dissociation

Tissue dissociation was performed as previously described in Lim et al. (Lim et al., 2022). Individual microtissues cultured on collagen coated-permeable membranes were washed with pre-warmed HBSS +Ca/Mg. The tissues were excised from the culture inserts and incubated for 15 min in pre-warmed cell recovery solution (354253, Corning, Tewksbury, MA). The mixture was pulse-agitated using a vortex mixer at 5 min intervals to promote tissue detachment. The tissues were subsequently peeled off from the membrane with fine point forceps under stereo microscope (Leica Microsystems, Wetzlar, Germany). The peeled tissues were placed into gentleMACS C-tube (130–093–237, Miltenyi Biotech, Bergisch Gladbach, Germany) containing the enzyme mix prepared according to protocols described in the human tumour dissociation kit (Miltenyi Biotec, Bergisch Gladbach, Germany). The tubes were then transferred into a gentleMACS octo dissociator with heater (Miltenyi Biotech, Bergisch Gladbach, Germany), and subjected to one cycle of ‘37C_m_LPDK_1′ program. Upon completion, the cell suspension was centrifuged at 1000 rpm for 5 min; the supernatant was removed, and the pellet was resuspended in 2 mL of HBSS +Ca/Mg. Total cell number was estimated using a haemocytometer and cell viability was determined as relative cell count, RCC). RCC is assessed by counting the total number of viable cells extracted from microtissues treated with the test substance and normalized against the total count of viable cells from tissues treated with the solvent control. Cytotoxicity was estimated by the formula“% Cytotoxicity = 100 – RCC”. Cytotoxicity can be readily inferred from the RCC data. The cells were then fixed for MN cytome scoring.

### Micronucleus cytome assay procedure

Treated microtissues were harvested and processed into single cell suspension as described. Cell samples were fixed in Carnoy's solution with 3 fixation steps in methanol/acetic acid 16:1, supplemented with 4 % formaldehyde at 1st fixation. The cell suspension was incubated in Carnoy's fixative at least overnight at 4°C to improve the quality of fixed cells. After overnight fixation the cell suspension was spread on microscope slides, dried, then stained with acridine orange and were immediately scored. One thousand cells were scored per criteria originally described by Tolbert et al. (Tolbert et al., 1992) and Thomas et al. (Thomas et al., 2009). One thousand total cells were scored per microtissue to determine the frequency of MN and cytome. The percentage of MN and cytome at each concentration of test chemical was compared with the vehicle (solvent) control. The results were generated from three independent experiments. Each experiment consisted of a single microtissue per dose. We refer readers to the publication of Lim et al. (Lim et al., 2022) for full details of the procedure and representative microphotographs that illustrate the different anomalies scored.

### Assay acceptance criteria

In order to compare the results of the RICyt assay we have implemented the following standard assay acceptance criteria:

1. Experiment should consist of a vehicle or solvent control (negative control), positive control and a minimum of three concentrations of test chemicals.
2. At least one microtissue per test concentration per experiment is included. Final results were deduced from three independent experiments to determine validity and reproducibility of the experimental trend and outcome.
3. Minimum of 1000 mononucleated cells were scored per microtissues.
4. Cells were exposed to the test chemical and sampled at a time equivalent to approximately one cell cycle length after the beginning of treatment (Lim et al., 2022).
5. The mean %MN for solvent control (negative controls) was within the 95 % upper confident limits based on Poisson distribution of the established historical negative control range (≤2.4 % in this study).
6. A positive result is defined when test chemicals show a statistically significant increase in %MN in at least for one of the test article concentrations (in relative to concurrent solvent control), and the results are outside the distribution of the historical negative control range (>2.4 % in this study). The MN frequency should also exhibit dose dependent response.
7. A negative result is defined when the criteria in step 6 are not met.
8. In the event of excessive cytotoxicity (≥50 % reduction in cell number relative to the solvent control) was noted, the sample were excluded from evaluation to avoid artefactual positive responses (OECD 2023).

### Calculation of the predictive capacity of the RICyt assay

The predictive parameters sensitivity, specificity and accuracy were calculated from 2 × 2 contingency tables. The sensitivity, specificity and accuracy of the assay were modelled using the following formulas:

**Table:** 

Sensitivity	(TP)/(TP+FN) × 100
Specificity	(TN)/(FP+TN) × 100
Accuracy	(TP+TN)/(TP+TN+FP+FN) × 100

where TP is true positive, TN is true negative, FN is false negative, and FP is false positive.

### Statistical analysis

All data are presented as means ± standard deviation of mean. One-way ANOVA and Tukey's post hoc test was used to determine the significance between groups and data was deemed significant if *p* < 0.05. All experiments were performed three times independently. Each experiment consisted of a single microtissue per dose unless otherwise indicated in figure legends. Poisson distribution is used to analyse the count distribution of mean MN number in the solvent controls when establishing historical negative range. 95 % upper confident limits of the distribution inform the highest acceptable value for the historical negative range.

## Results

### Historical negative range data generation

**Fig. 1:**
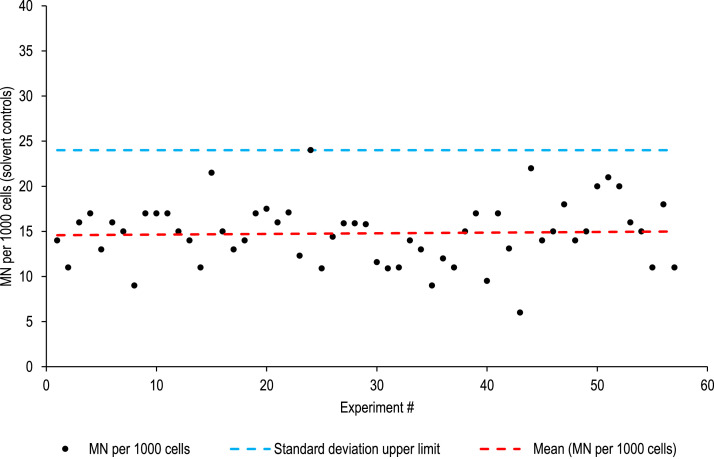
Historical negative range for solvent controls used in RICyt assay. Micronuclei, MN per 1000 cells, (black dots) in individual EpiIntestinal™ microtissues treated with solvent control (DMSO or H_2_O) for 10 days. Each black dot represents data from a single treated tissues. Red- dashed line denotes historical mean value. Blue-dashed line denotes 95% upper confident limits based on Poisson distribution.

Historical negative controls range are essential for data quality and analysis of genetic toxicity assay results (OECD 2023). The negative control data are typically used for comparison with the concurrent control data as part of the assay acceptance criteria. Here, the laboratory-specific historical reference for solvent control were derived to determine experimental variability and used for subsequent data interpretation. The assay was performed using solvent control-treated microtissues. This study has identified the range of historical negative control data collected from concurrent solvent controls among 53 independent experiments conducted previously. The reported mean is 1.48 %. The lowest acceptable value is calculated at 0.8 % while the highest is 2.4 % based on 95 % confident limits of Poisson distribution. The historical negative/solvent control data distribution are shown in Fig. 1. Most of the data from concurrent solvent controls were included, excluding only those with technical error. The data range has surpassed the preferred recommended independent experiment number (*i.e.* 20) and scored from at least 2000 cells from two microtissues per experiment in order to provide an acceptable range for historical control databases (OECD 2023; Lovell et al., 2018; Bemis et al., 2016; Hayashi et al., 2011). The data were obtained under the identical experimental conditions and scoring criteria as test groups. In the RICyt assay, a chemical is determined to be a positive genotoxicant when a statistically significant increase in MN frequency is observed in at least one concentration over historical negative controls range. Experiments where the concurrent negative control data deviate from the historical control data were not discounted unless a solid scientific justification was identified.

### Genotoxins

**Fig. 2:**
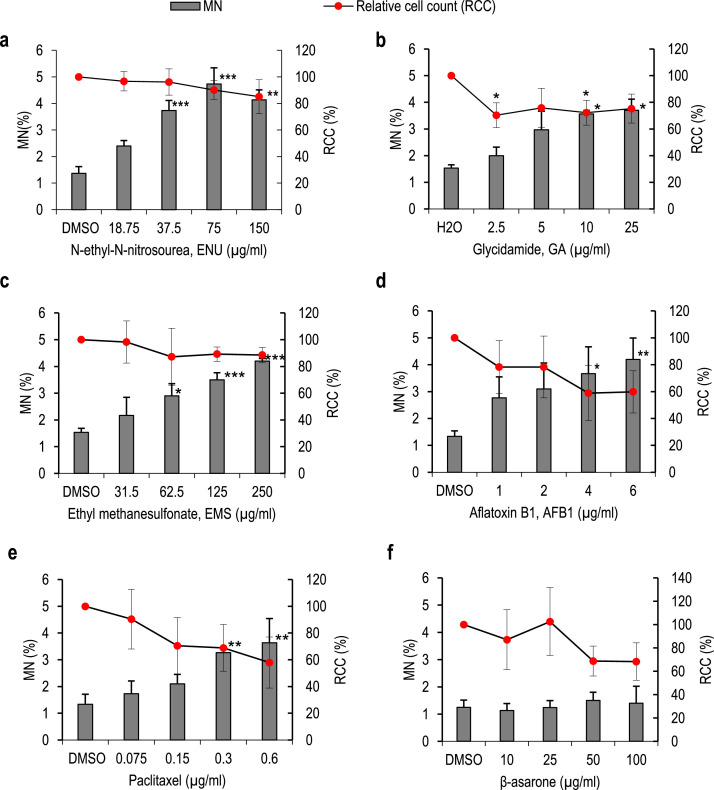
Percentage of MN in mononucleted cells and RCC of known genotoxins tested in RICyt assay. EpiIntestinal™ microtissues were exposed to test articles and scored according to the criteria described in “Materials and methods”. One thousand total cells were scored per microtissue to determine the frequency of MN induction. The results from three independent experiments are shown. Each experiment consisted of a single microtissue per test dose. *p < 0.05, **p < 0.01 and ***p < 0.001.

N-Ethyl-N-nitrosourea, ENU, was tested up to 150 µg/ml and no concentration achieved cytotoxicity >15 %. All four tested concentrations resulted in statistically significant increases in MN in a dose dependent manner, relative to solvent control. The percentage induction was outside the distribution of the historical negative control range. There was also non-significant increase in karyorrhectic cells from the cytome analysis (Fig. 2a and Supplemental Table 1). Glycidamide, GA, was tested up to 25 µg/ml and produced mild cytotoxicity of approximately 25–30 % in tested concentrations. GA showed significant increases in the %MN that exceeded the 95 % confident limit of the historical negative control data at multiple concentrations. Non-significant increase in karyorrhectic cells produced (Fig. 2b and Supplemental Table 1). Ethyl methanesulfonate, EMS, induced a clear and dose-dependent increase in %MN at multiple doses. There was no observable cytotoxicity induced at tested doses (Fig. 2c and Supplemental Table 1). Aflatoxin B1, AFB1, induced significant and dose-dependent formation of MN in the EpiIntestinal™ microtissues, with corresponding significant loss in cell viability (∼ 40 % at top two concentrations) (Fig. 2d and Supplemental Table 1) after repeated exposure. Paclitaxel belongs to the “Taxane” group of chemotherapeutic agents (a class of diterpenes) that has been used in the treatment of various cancers (Jayakody et al., 2018). Following ten-day exposure, our data revealed that EpiIntestinal™ microtissues were susceptible to the effect of Paclitaxel, which resulted in statistically significant increase in %MN with corresponding decrease in cell viability at the top two exposed doses. This was accompanied by a non-significant but dose dependent increase in karyorrhectic cell population in the exposed tissues (Fig. 2e and Supplemental Table 1).

### Non-genotoxins

**Fig. 3:**
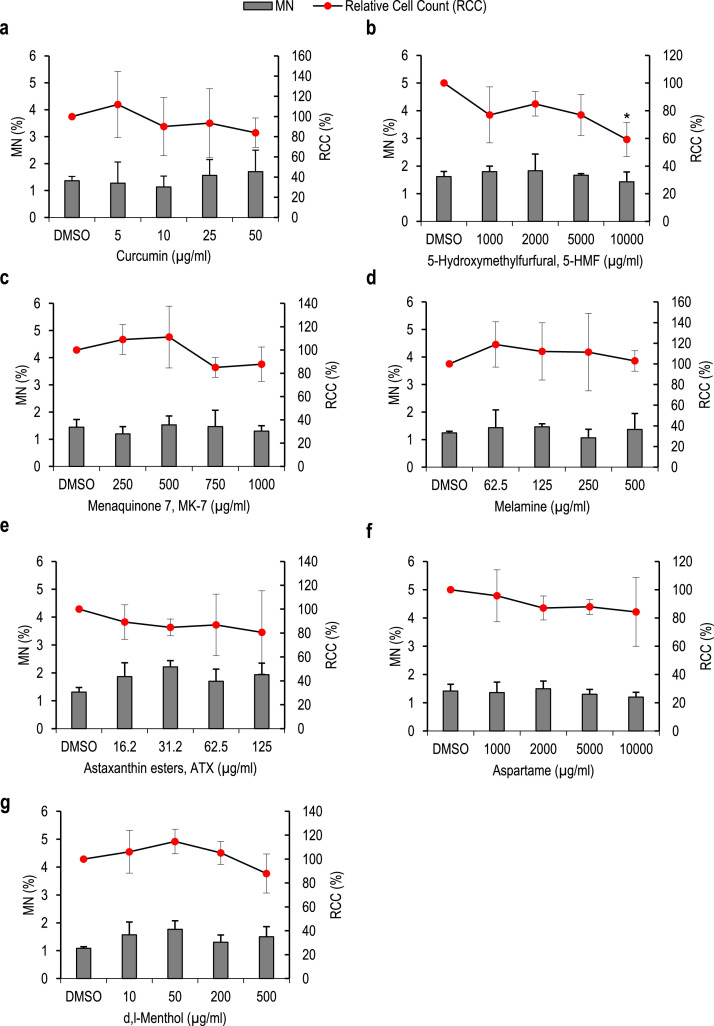
Percentage of MN in mononucleted cells and RCC of non-DNA-reactive chemicals (including non-genotoxins) tested in RICyt assay. EpiIntestinal™ microtissues were exposed to test chemicals and scored according to the criteria listed in “Materials and methods”. One thousand total cells were scored per microtissue to determine the frequency of MN induction. The results from three independent experiments are shown. Each experiment consisted of a single microtissue per test dose.

From our experimental outcome, there was no significant formation of MN following curcumin exposure though 15–20 % cytotoxicity observed at higher doses. The exposure also resulted in an increase in karyorrhectic cells (Fig. 3a and Supplemental Table 2). It was recommended that the guidance limit for top test concentration for any chemicals should be at 2000 µg/ml or highest possible depending on cytotoxicity and test material solubility (OECD 2023). Curcumin was insoluble beyond 50 µg/ml and therefore the top test concentration was determined as such. 5-Hydroxymethylfurfural, 5-HMF, exposure showed that the %MN induction was consistently low without exceeding the historical negative range, even with excessively high treatment dose up to 10,000 µg/ml. Cytotoxicity was observed with increasing doses of 5-HMF. There was an increase in karyorrhectic cell population though not statistically significant (Fig. 3b and Supplemental Table 2). Menaquinone 7, MK-7, showed no significant MN induction nor cytotoxicity following 10-day exposure in our investigation. The formation of other nuclear anomalies was not detected (Fig. 3c and Supplemental Table 2). The highest test concentration for MK-7 was limited at 1000 µg/ml due to insolubility of MK-7 in solvent above this concentration. Melamine was tested through our model. No genotoxicity nor cytotoxicity were detected after exposure (Fig. 3d and Supplemental Table 2). All the %MN values generated were not significant and below the historical negative range. The top test concentration of melamine was limited to 500 µg/ml due to test material insolubility above this concentration. Astaxanthin esters, ATX, did not induce significant increase in %MN, though with subtle reduction in cell viability (∼20 %) and a non-significant increase in karyorrhectic cell population (Fig. 3e and Supplemental Table 2), following 10-day exposure of EpiIntestinal™ microtissues. The top test concentration of ATX was limited to 125 µg/ml due to test material insolubility above this concentration. Aspartame did not produce significant %MN and cytotoxicity in our model within the tested range, even at the excessive highest concentration at 10,000 µg/ml (Fig. 3f and Supplemental Table 2). D,L-Menthol through our study showed that this compound is not genotoxic as the exposure produced low level of MN which fell within 95 % confident limit of the historical negative control. There was no significant impact on cell viability as well (Fig. 3g and Supplemental Table 2). The limiting top test concentration was determined at 500 µg/ml due to test material insolubility above this concentration.

### Genotoxin with false negative outcome

**Fig. 4:**
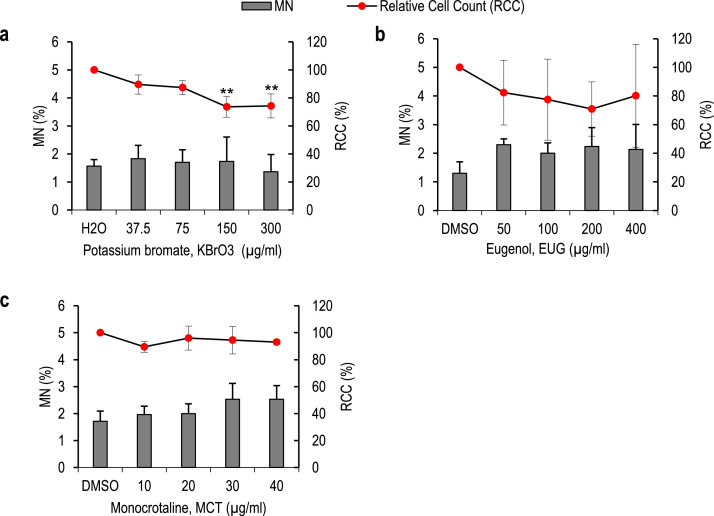
Percentage of MN in mononucleted cells and RCC of chemicals with equivocal or unexpected results tested in RICyt assay. EpiIntestinal™ microtissues were exposed to test chemicals and scored according to the criteria listed in “Materials and methods”. One thousand total cells were scored per microtissue to determine the frequency of MN induction. The results from three independent experiments are shown. Each experiment consisted of a single microtissue per test dose. **p < 0.01.

Potassium bromate, KBrO_3_, in this study, did not induce any significant increase in %MN and other nuclear anomalies within the tested concentrations. The %MN formation falls within the historical negative control range. Though, approximately 27 % cytotoxicity were induced at top two concentrations (Fig. 4a and Supplemental Table 3). The top test concentration was limited to 300 µg/ml, beyond which >50 % cell death observed in the treated microtissues (data not shown).

### Genotoxin with negative outcome

β-asarone induced low level of %MN near to the level induced by the DMSO solvent control. Increases in cytotoxicity at the top two doses observed (Fig. 2f and Supplemental Table 1). Monocrotaline, MCT, induced a dose-dependent and non-significant increase in %MN in the exposed EpiIntestinal™ microtissues without obvious cytotoxicity (Fig. 4c and Supplemental Table 3). Though the %MN increment is lower (2.53 %) compared to other genotoxins tested.

### Chemicals with equivocal outcome

Our experimental data showed the value of %MN induced by EUG were very close to the established 95 % upper confident limits and cytotoxicity observed in the tested doses. There was also consistent increase in karyorrhectic cell population (Fig. 4b and Supplemental Table 3).

### Predictive capacity of the RICyt assay

Results of all chemicals tested in the RICyt assay are summarized in Table 1, Table 2 and were compared with existing published *in vitro* and *in vivo* MN status of each chemical. Five out of seven known MN positive genotoxins were identified as true positives. All of the MN negative genotoxin and known non-genotoxins produced true negative data range. One false negative (KBrO_3_) and one equivocal result (EUG) was produced. MCT produced MN negative results infer the tissue specificity possessed by RICyt assay. Applying the approach established by our team (described in the material and methods sections) reveals a sensitivity of 83.3 % and a specificity of 100 %. The resulted overall accuracy is 93.3 % (Table 3).

**Table 3:** Sensitivity and specificity of RICyt assay. Fifteen total test articles was included in the final analysis. Equivocal test article (Eugenol) was not included in the calculation.

	MN +ve	MN -ve
MN +ve	5 True positive (TP)	0 False positive (FP)
MN -ve	1 False negative (FN)	9 True negative (TN)

Sensitivity	83.3	
Specificity	100	
Accuracy	93.3	

## Discussion

The aim of this study was to evaluate the qualification of RICyt assay protocol previously developed by our team (Lim et al., 2022) for site-of-contact genotoxic hazard identification and establish associated acceptance criteria for the assay. The RICyt assay was developed with a commercial 3D EpiIntestinal™ platform of reconstructed microtissues. The 3D EpiIntestinal™ microtissues retain properties akin to native small intestines, which includes tissue architecture, heterogenous cell types (enterocytes, paneth cells, M cells, tuft cells and intestinal stem cells), tissue polarization, barrier functions, adsorption and biochemical signature. This evaluation is a small-scale investigation ahead of formal assay validation. The data suggested that RICyt assay produced reliable predictivity and a promising technology capable of supporting site-of-contact genetic hazard identification.

This evaluation showed that RICyt assay can differentiate genotoxins from non-genotoxins. To investigate the qualification of RICyt assay in genotoxic hazard identification, we challenged the platform with test chemicals with various mechanism of action. When exposed to ECVAM recommended positive materials, ENU, EMS, Paclitaxel, and genotoxic metabolite GA, true positive results were generated in the test system. These test materials are highly recognized materials with well-established genotoxicity evidence in various standard and non-standard *in vitro* and *in vivo* genetic toxicity tests. When exposed to standard non-genotoxins, curcumin, 5-HMF, MK-7, melamine, ATX, aspartame and D,L-Menthol, clear negative results were detected.

The RICyt assay has also showed the ability to detect the genotoxicants with different genotoxic mechanisms. In this study and our previous publication (Lim et al., 2022), exposure to clastogens (ENU, EMS, GA and mitomycin C) and aneugens (Paclitaxel and vinblastine) resulted in positive induction of MN in the RICyt platform. It was reported earlier that EpiIntestinal™ microtissues are partially metabolic competent (Ayehunie et al., 2018; Cui et al., 2020). When the 3D reconstructed intestinal tissues was tested with progenotoxin such as AFB1 (this study) and benzo(a)pyrene (Lim et al., 2022) the genotoxicity was successfully detected without the use of an exogenous metabolic system. The observation suggests EpiIntestinal™ microtissues possess sufficient metabolic competency. These evidence infer the assay is able to detect clastogenic, aneugenic and progenotoxic test substances.

β-asarone is an *in vivo* MN negative genotoxin. Genotoxicity is largely due to the mutagenic effect of β-asarone on targeted models. This material is mainly metabolized by CYP3a4 in order to release metabolites such as highly unstable and reactive epoxides, which are genotoxic (Kobets et al., 2016; Cartus et al., 2015). Contradictory *in vitro* MN data had been reported for β-asarone since clastogenic effects have been observed in some *in vitro* models in the presence or absence of metabolic activation (Abel, 1987; Chellian et al., 2017) but the same effect was not detected in other *in vitro* MN study (Devi and Babu, 2015). No data on the *in vivo* genotoxicity have been identified. Based on our observation and literature review, EpiIntestinal™ microtissues is CYP3a4 competent and able to metabolize β-asarone. However, it was not conclusive in treated EpiIntestinal™ microtissues whether β-asarone epoxide metabolites were synthesized through the similar metabolism or whether the metabolism produced sufficient epoxide metabolites to induce MN. Our data revealed no significant MN formation in the treated tissues, which corresponds to their *in vivo* MN negative status.

KBrO_3_ exposure produced false negative results through this platform. KBrO_3_ has been classified as IARC Group 2B carcinogen (Shanmugavel et al., 2020). Based on our observation, KBrO_3_ is classified as negative in RICyt assay. This result is opposite to the known genotoxic status of KBrO_3._ Hence, KBrO_3_ was placed under the false negative category. Our team had previously identified KBrO_3_ as weak genotoxic positive in comet assay developed with EpiIntestinal™ microtissues (RICom) at high doses following 48 h exposure (up to 741 µg/ml and 40 % cell loss) (Hughes et al., 2023). When repeating the assay for 10 days with RICyt assay protocol at the same doses, the microtissues would experience >50 % of cell death. Hence, lower test concentrations were selected (up to 300 µg/ml). While at lower doses, no significant MN formation detected. When higher test concentration 500 µg/ml was used in trial experiments, there still wasn't any significant MN induction (data not shown). Study had shown that KBrO_3_ induces oxidative DNA damage only at high enough doses but not at low doses (Wei et al., 2009). This could serve as a possible explanation to our observation of the contradicting results between MN and comet assay using the same microtissue platform. KBrO_3_ was found to have impact on small intestine in *in vivo* studies (Aoki et al., 2021). The opposing outcome in our system suggests the genotoxicity of KBrO_3_ in term of MN formation not detected in EpiIntestinal™ microtissues due to the above-mentioned limiting factor. However, KBrO_3_ induced DNA fragmentations can still be detected by RICom assay.

MCT are toxic secondary metabolites exclusively biosynthesised by plants. These metabolites are grouped under IARC Group 2B carcinogen. The metabolic activation of MCT occurs via dehydrogenation primarily, by CYP3A4, CYP2B6 and CYP2C to form dehydromonocrotaline (DHM), which is highly reactive and toxic metabolites (Yao et al., 2014), responsible for MCT-induced genotoxicity. Poisoning caused by MCT is associated with liver, kidney and pulmonary toxicity. Limited evidence has been reported on the toxic effect of MCT on GI tract. Caco-2 permeability measurements show MCT have moderate gut permeability, affecting tight junction proteins but no genotoxic evidence reported (Schrenk et al., 2022). Though MCT exposure resulted in slight increases in %MN but it was not statistically significant and only met partial acceptance criteria for positive genotoxicants via RICyt platform. This could indicate the site-of-contact effect of MCT was absent in the intestinal model. Hence, MCT is classified as negative from our observation.

EUG produced equivocal outcome in this assay. It has been reported as IARC Group 3 substance and found to have equivocal genotoxic profile under certain conditions. EUG is unlikely to be genotoxic at exposures that do not result in cytotoxicity (European Food Safety 2012). But EUG is genotoxic positive at cytotoxic concentrations *in vitro* and at very high doses *in vivo* (European Food Safety 2012). EUG has been identified as equivocal in our study because the resulted MN values were very close the established upper confident limits (2.4 %). One possible underlying reason for EUG induced MN and cytotoxicity in our model is the fact that EUG could inhibit topoisomerase II activity (Maralhas et al., 2006). This leads to the formation of single or double strand breaks to the DNA and cells will verge themselves into apoptosis mechanisms (Le Hégarat et al., 2014). Topoisomerase II is more strongly expressed in rapidly dividing cells and tissues such as skin, lymph nodes, and bone marrow (Uhlén et al., 2015). The EpiIntestinal™ microtissues consists of both rapidly dividing stem cells located at the intestinal crypts and non-dividing differentiated cells at villus. It is hypothesized that topoisomerase II activity expressed by the intestinal stem cells were inhibited by EUG and resulted in our observation. Though EUG is pro-oxidative at high concentrations due to the formation of catechols after metabolic reactions, the EpiIntestinal™ microtissues lack of CYP2E1 to activate oxidative activity of catechol (Cui et al., 2020). Thus, the contribution of catechol to EUG-induced MN can be excluded.

The GI tract is a major site of exposure to ingested genotoxicants. However, the genotoxic effect to the GI tract has largely been spared in the past. Potential site-of-contact genotoxic risk is currently not yet addressed in *in vitro* genotoxicity assays. Recommendations had been made to include site-of-contact genotoxicity assessment as follow-up of *in vitro* results from the basic battery tests before embarking on any necessary *in vivo* testing (More et al., 2019). The site-of-contact effect is largely depending on route of administration. Current *in vitro* MN assay doesn't produce sufficient local contact information or any tissue specificity. At present, validated *in vivo* bone marrow and peripheral blood MN assay answer systemic genotoxicity rather than local effect where site-of-contact occurs. It is possible that local concentrations may be higher than systemic concentrations and the local genotoxicity might be overlooked (More et al., 2019). Though the *in vivo* gut MN assay was developed by other research groups but no validation or follow-up on the assay predictivity (Ohyama et al., 2023; Vanhauwaert et al., 2001). The RICyt assay is the first assay that offers site-of-contact genotoxicity identification of ingested substances. The 3D Epiintestinal™ microtissues which express several vital morphological, molecular and biochemical features of human small intestine, made *in vitro* site-of-contact and tissue specific genotoxicity assessment of ingested substances achievable.

The Caco-2 cell line has been widely used in the micronucleus assay and produces decent preliminary predictions (Le Hégarat et al., 2012; Pinto-Silva et al., 2006). However, it is a cancer cell line and lacks p53 expression (Ray et al., 2011; Thant et al., 2008). Caco-2 cells express certain features of intestinal epithelium upon differentiation (Wanes et al., 2021; Pageot et al., 2000)**.** Cellular proliferation is essential for micronuclei formation (OECD 2023). Caco-2 cells maintain a somewhat uncontrolled proliferation rate due to their cancerous origin, unlike normal intestinal epithelial cells with tightly regulated cell proliferation (Wanes et al., 2021; Pageot et al., 2000). The EpiIntestinal microtissues are reconstructed from normal intestinal epithelial cells, recapitulating plenty structural, molecular and functional aspects of the human small intestine (Ayehunie et al., 2018). The intestinal villus of EpiIntestinal microtissues provides a larger surface area for test chemicals uptake and adsorption in a similar manner as the human small intestine as opposed to Caco-2 cells which are most easily raised as 2D monolayer cultures. In addition, Caco-2 is a colonic cell line, however, the majority of ingested substances are absorbed in the small intestine making it an important site for contact and interaction of ingested substances and an important site for hazard identification. It is important to complement simpler 2D Caco-2 based assays with more physiologically relevant assays like the RICyt assay to better inform human risk assessment.

Our investigation reveals RICyt assay demonstrated decent sensitivity. However, the magnitude of MN% increment is smaller (*i.e.* 2–3.5-fold) compared to standard *in vitro* and *in vivo* MN assay. The magnitude of increment is limited by three major factors: 1) intestinal cell shedding, 2) spontaneous background frequency of micronucleated cell and 3) lower percentage of actively proliferating cells. The EpiIntestinal™ microtissue maintains a relatively high rate of cellular equilibrium which consists of active cell growth, migration, cell death and cell shedding, similar with native human small intestine (Bullen et al., 2006). The shed cells were removed by HBSS rinses during routine media change and article treatment. The active cell shedding likely resulted in the under-detection of MN. The spontaneous background frequency of MN in this model is comparatively higher than most *in vivo* or the 3D skin MN assay. This could be due to the *in vitro* culture condition that resulted in higher background MN compared to *in vivo* condition. When compared to the skin MN assay which used cytochalasin B treatment and focused only on proliferative cells, we took consideration of total cell population. Internal trial experiments revealed EpiIntestinal™ microtissue consists of approximately 5–10 % actively proliferating cells (data not shown). Cytochalasin B also impacts intestinal tissue tight junction proteins creating a ‘leaky’ epithelial barrier and is likely unsuitable for application with intestinal cells and models (Ma et al., 2000). Hence, the decision was made earlier to focus on MN cytome instead. The standard EpiIntestinal™ model is reconstructed using epithelial cells extracted from the ileum section of the gut. Though the organotypic tissues from the duodenum and jejunum segments of the small intestine have also been developed separately (Markus et al., 2021). The responses evidenced from EpiIntestinal™ model can be used as an indication of the chemical exposure outcome that might happen to the ileum but not representative for outcome prediction for the entire small intestine and GI tract.

## Conclusion

In this study, we have presented a small-scale qualification study of the RICyt assay to predict genotoxicity of ingestible genotoxic agents. Our investigation revealed the RICyt assay possesses decent predictive power for genetic hazard identification and first *in vitro* tool valuable for site-of-contact genotoxicity assessment for oral or GI route of exposure. However, the scoring approach can be further optimized in future study to increase the magnitude of MN increment of the assay. We plan to focus on the MN in the proliferative cells and eliminate differentiated cells when scoring. One can do so by recognizing the nuclear size and intensity of the mononucleated cells since proliferative cells come with bigger and less intense nucleus. Whilst differentiated cells have smaller and more intense nucleus (Bolognesi and Fenech, 2019). Another possible approach is to fluorescently stain with proliferative cell marker and only focus on the stain-positive cells during the scoring process. Investigative studies with wider range of food-relevant test materials should also be conducted to further assess the assay protocol validity and reproducibility. We believe this study has paved a promising future path for RICyt assay to be employed in the early stages to identify the genotoxic potential of ingestible materials (especially complex and novel food ingredients and tecnologies) and subsequently to design out the risk.

## Ethics approval

The manuscript does not report any clinical or patient data.

## Research data for this article

Available upon request

## CRediT authorship contribution statement

**Hui Kheng Lim:** Writing – review & editing, Writing – original draft, Validation, Project administration, Methodology, Investigation, Formal analysis, Data curation, Conceptualization. **Christopher Owen Hughes:** Writing – review & editing, Validation, Methodology, Investigation, Formal analysis. **Timothy Landry:** Writing – review & editing. **Choon Wee Joseph Tan:** Validation, Methodology, Formal analysis. **Seyoum Ayehunie:** Writing – review & editing. **Benjamin Paul Chapman Smith:** Writing – review & editing, Validation, Supervision, Conceptualization.

## Declaration of competing interest

The authors declare the following financial interests/personal relationships which may be considered as potential competing interests:

Lim Hui Kheng reports financial support, administrative support, article publishing charges, and equipment, drugs, or supplies were provided by FRESH NTU. Lim Hui Kheng reports a relationship with FRESH NTU that includes: employment. If there are other authors, they declare that they have no known competing financial interests or personal relationships that could have appeared to influence the work reported in this paper.

## Data Availability

Data will be made available on request.

## Appendix Supplementary materials

Image, application 1

Image, application 2

Image, application 3

Image, application 4
