# Supplementary material for: Qualification of the human Reconstructed Intestine Micronuclei Cytome assay for site-of-contact genotoxic hazard identification
Source: NAM J. 2025 Mar 28;1:100015. doi: 10.1016/j.namjnl.2025.100015 (PMC13289030; doi:10.1016/j.namjnl.2025.100015)
Supplement: Supplementary file 1 [file mmc1.docx]

**Glossary**

| RICyt | Reconstructed Intestine Micronuclei Cytome |
| --- | --- |
| MN | Micronuclei |
| GI | Gastrointestinal |
| NAMs | New Approach Methodologies |
| 2D | Two dimensional |
| 3D | Three dimensional |
| MoA | Mechanism of action; |
| CYP | Cytochrome P450 |
| CA | Chromosomal aberrations |
| HPRT | Hypoxanthine-guanine phosphoribosyl transferase |
| UDS | Unscheduled DNA synthesis |
| SCE | Sister chromatid exchange |
| MLA | Mouse lymphoma |
| SULT | Sulfotransferase |
| UDP | Uridine-5'-diphosphate |
| UGT | UDP glucuronosyltransferases |
| ENU | N-ethyl-N-nitrosourea |
| GA | Glycidamide |
| AFB1 | Aflatoxin B1 |
| EMS | Ethyl methanesulfonate |
| Buds | Nuclear buds, |
| Mono | Mononucleated cells |
| BN | Binucleated cells |
| CC | Condensed chromatin |
| KR | Karyorrhexis |
| PY | Pyknosis |
| KL | Kkaryolysis; |
| 5-HMF | 5-Hydroxymethylfurfural |
| MK-7 | Menaquinone 7 |
| ATX | Astaxanthin; |
| KBrO3 | Potassium bromate |
| EUG | Eugenol |
| MCT | Monocrotaline |
| HBSS | Hanks Balanced Salts Solution |
| DMSO | Dimethylsulfoxide |
| H_2_O | Water |
| β | Beta |
| TP | True positive |
| TN | True negative |
| FN | False negative |
| FP | False positive |
| RICom | Reconstructed human intestinal comet |
| IARC | International Agency for Research on Cancer |
| DHM | Dehydromonocrotaline |
