# Supplementary material for: Qualification of the human Reconstructed Intestine Micronuclei Cytome assay for site-of-contact genotoxic hazard identification
Source: NAM J. 2025 Mar 28;1:100015. doi: 10.1016/j.namjnl.2025.100015 (PMC13289030; doi:10.1016/j.namjnl.2025.100015)
Supplement: Supplementary file 2 [file mmc2.pptx]

## Slide 1
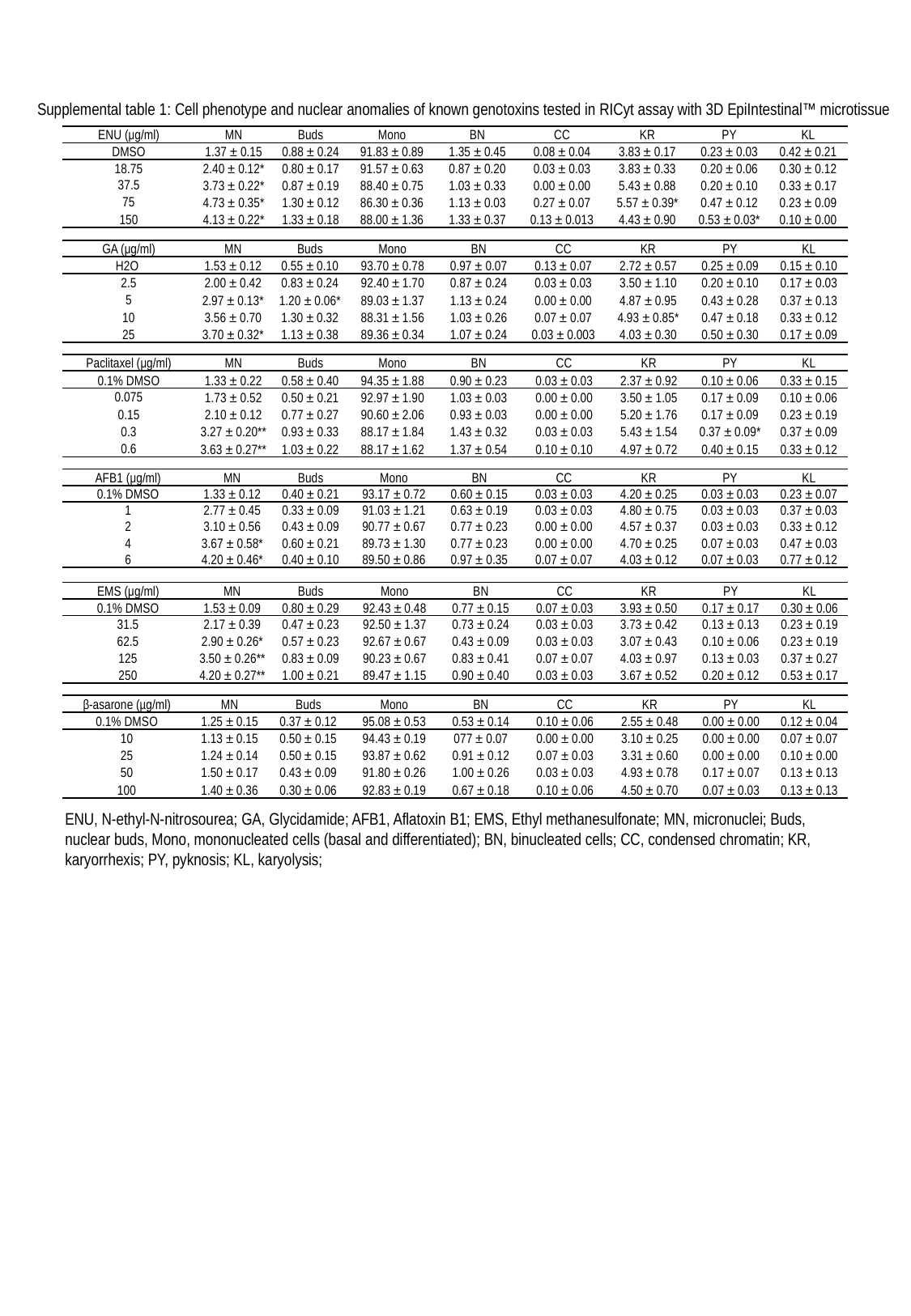

Supplemental table 1: Cell phenotype and nuclear anomalies of known genotoxins tested in RICyt assay with 3D EpiIntestinal™ microtissue
| ENU (μg/ml) | MN | Buds | Mono | BN | CC | KR | PY | KL |
| --- | --- | --- | --- | --- | --- | --- | --- | --- |
| DMSO | 1.37 ± 0.15 | 0.88 ± 0.24 | 91.83 ± 0.89 | 1.35 ± 0.45 | 0.08 ± 0.04 | 3.83 ± 0.17 | 0.23 ± 0.03 | 0.42 ± 0.21 |
| 18.75 | 2.40 ± 0.12\* | 0.80 ± 0.17 | 91.57 ± 0.63 | 0.87 ± 0.20 | 0.03 ± 0.03 | 3.83 ± 0.33 | 0.20 ± 0.06 | 0.30 ± 0.12 |
| 37.5 | 3.73 ± 0.22\* | 0.87 ± 0.19 | 88.40 ± 0.75 | 1.03 ± 0.33 | 0.00 ± 0.00 | 5.43 ± 0.88 | 0.20 ± 0.10 | 0.33 ± 0.17 |
| 75 | 4.73 ± 0.35\* | 1.30 ± 0.12 | 86.30 ± 0.36 | 1.13 ± 0.03 | 0.27 ± 0.07 | 5.57 ± 0.39\* | 0.47 ± 0.12 | 0.23 ± 0.09 |
| 150 | 4.13 ± 0.22\* | 1.33 ± 0.18 | 88.00 ± 1.36 | 1.33 ± 0.37 | 0.13 ± 0.013 | 4.43 ± 0.90 | 0.53 ± 0.03\* | 0.10 ± 0.00 |
| GA (μg/ml) | MN | Buds | Mono | BN | CC | KR | PY | KL |
| --- | --- | --- | --- | --- | --- | --- | --- | --- |
| H2O | 1.53 ± 0.12 | 0.55 ± 0.10 | 93.70 ± 0.78 | 0.97 ± 0.07 | 0.13 ± 0.07 | 2.72 ± 0.57 | 0.25 ± 0.09 | 0.15 ± 0.10 |
| 2.5 | 2.00 ± 0.42 | 0.83 ± 0.24 | 92.40 ± 1.70 | 0.87 ± 0.24 | 0.03 ± 0.03 | 3.50 ± 1.10 | 0.20 ± 0.10 | 0.17 ± 0.03 |
| 5 | 2.97 ± 0.13\* | 1.20 ± 0.06\* | 89.03 ± 1.37 | 1.13 ± 0.24 | 0.00 ± 0.00 | 4.87 ± 0.95 | 0.43 ± 0.28 | 0.37 ± 0.13 |
| 10 | 3.56 ± 0.70 | 1.30 ± 0.32 | 88.31 ± 1.56 | 1.03 ± 0.26 | 0.07 ± 0.07 | 4.93 ± 0.85\* | 0.47 ± 0.18 | 0.33 ± 0.12 |
| 25 | 3.70 ± 0.32\* | 1.13 ± 0.38 | 89.36 ± 0.34 | 1.07 ± 0.24 | 0.03 ± 0.003 | 4.03 ± 0.30 | 0.50 ± 0.30 | 0.17 ± 0.09 |
| Paclitaxel (μg/ml) | MN | Buds | Mono | BN | CC | KR | PY | KL |
| --- | --- | --- | --- | --- | --- | --- | --- | --- |
| 0.1% DMSO | 1.33 ± 0.22 | 0.58 ± 0.40 | 94.35 ± 1.88 | 0.90 ± 0.23 | 0.03 ± 0.03 | 2.37 ± 0.92 | 0.10 ± 0.06 | 0.33 ± 0.15 |
| 0.075 | 1.73 ± 0.52 | 0.50 ± 0.21 | 92.97 ± 1.90 | 1.03 ± 0.03 | 0.00 ± 0.00 | 3.50 ± 1.05 | 0.17 ± 0.09 | 0.10 ± 0.06 |
| 0.15 | 2.10 ± 0.12 | 0.77 ± 0.27 | 90.60 ± 2.06 | 0.93 ± 0.03 | 0.00 ± 0.00 | 5.20 ± 1.76 | 0.17 ± 0.09 | 0.23 ± 0.19 |
| 0.3 | 3.27 ± 0.20\*\* | 0.93 ± 0.33 | 88.17 ± 1.84 | 1.43 ± 0.32 | 0.03 ± 0.03 | 5.43 ± 1.54 | 0.37 ± 0.09\* | 0.37 ± 0.09 |
| 0.6 | 3.63 ± 0.27\*\* | 1.03 ± 0.22 | 88.17 ± 1.62 | 1.37 ± 0.54 | 0.10 ± 0.10 | 4.97 ± 0.72 | 0.40 ± 0.15 | 0.33 ± 0.12 |
| AFB1 (μg/ml) | MN | Buds | Mono | BN | CC | KR | PY | KL |
| --- | --- | --- | --- | --- | --- | --- | --- | --- |
| 0.1% DMSO | 1.33 ± 0.12 | 0.40 ± 0.21 | 93.17 ± 0.72 | 0.60 ± 0.15 | 0.03 ± 0.03 | 4.20 ± 0.25 | 0.03 ± 0.03 | 0.23 ± 0.07 |
| 1 | 2.77 ± 0.45 | 0.33 ± 0.09 | 91.03 ± 1.21 | 0.63 ± 0.19 | 0.03 ± 0.03 | 4.80 ± 0.75 | 0.03 ± 0.03 | 0.37 ± 0.03 |
| 2 | 3.10 ± 0.56 | 0.43 ± 0.09 | 90.77 ± 0.67 | 0.77 ± 0.23 | 0.00 ± 0.00 | 4.57 ± 0.37 | 0.03 ± 0.03 | 0.33 ± 0.12 |
| 4 | 3.67 ± 0.58\* | 0.60 ± 0.21 | 89.73 ± 1.30 | 0.77 ± 0.23 | 0.00 ± 0.00 | 4.70 ± 0.25 | 0.07 ± 0.03 | 0.47 ± 0.03 |
| 6 | 4.20 ± 0.46\* | 0.40 ± 0.10 | 89.50 ± 0.86 | 0.97 ± 0.35 | 0.07 ± 0.07 | 4.03 ± 0.12 | 0.07 ± 0.03 | 0.77 ± 0.12 |
| EMS (μg/ml) | MN | Buds | Mono | BN | CC | KR | PY | KL |
| --- | --- | --- | --- | --- | --- | --- | --- | --- |
| 0.1% DMSO | 1.53 ± 0.09 | 0.80 ± 0.29 | 92.43 ± 0.48 | 0.77 ± 0.15 | 0.07 ± 0.03 | 3.93 ± 0.50 | 0.17 ± 0.17 | 0.30 ± 0.06 |
| 31.5 | 2.17 ± 0.39 | 0.47 ± 0.23 | 92.50 ± 1.37 | 0.73 ± 0.24 | 0.03 ± 0.03 | 3.73 ± 0.42 | 0.13 ± 0.13 | 0.23 ± 0.19 |
| 62.5 | 2.90 ± 0.26\* | 0.57 ± 0.23 | 92.67 ± 0.67 | 0.43 ± 0.09 | 0.03 ± 0.03 | 3.07 ± 0.43 | 0.10 ± 0.06 | 0.23 ± 0.19 |
| 125 | 3.50 ± 0.26\*\* | 0.83 ± 0.09 | 90.23 ± 0.67 | 0.83 ± 0.41 | 0.07 ± 0.07 | 4.03 ± 0.97 | 0.13 ± 0.03 | 0.37 ± 0.27 |
| 250 | 4.20 ± 0.27\*\* | 1.00 ± 0.21 | 89.47 ± 1.15 | 0.90 ± 0.40 | 0.03 ± 0.03 | 3.67 ± 0.52 | 0.20 ± 0.12 | 0.53 ± 0.17 |
| β-asarone (µg/ml) | MN | Buds | Mono | BN | CC | KR | PY | KL |
| --- | --- | --- | --- | --- | --- | --- | --- | --- |
| 0.1% DMSO | 1.25 ± 0.15 | 0.37 ± 0.12 | 95.08 ± 0.53 | 0.53 ± 0.14 | 0.10 ± 0.06 | 2.55 ± 0.48 | 0.00 ± 0.00 | 0.12 ± 0.04 |
| 10 | 1.13 ± 0.15 | 0.50 ± 0.15 | 94.43 ± 0.19 | 077 ± 0.07 | 0.00 ± 0.00 | 3.10 ± 0.25 | 0.00 ± 0.00 | 0.07 ± 0.07 |
| 25 | 1.24 ± 0.14 | 0.50 ± 0.15 | 93.87 ± 0.62 | 0.91 ± 0.12 | 0.07 ± 0.03 | 3.31 ± 0.60 | 0.00 ± 0.00 | 0.10 ± 0.00 |
| 50 | 1.50 ± 0.17 | 0.43 ± 0.09 | 91.80 ± 0.26 | 1.00 ± 0.26 | 0.03 ± 0.03 | 4.93 ± 0.78 | 0.17 ± 0.07 | 0.13 ± 0.13 |
| 100 | 1.40 ± 0.36 | 0.30 ± 0.06 | 92.83 ± 0.19 | 0.67 ± 0.18 | 0.10 ± 0.06 | 4.50 ± 0.70 | 0.07 ± 0.03 | 0.13 ± 0.13 |
ENU, N-ethyl-N-nitrosourea; GA, Glycidamide; AFB1, Aflatoxin B1; EMS, Ethyl methanesulfonate; MN, micronuclei; Buds, nuclear buds, Mono, mononucleated cells (basal and differentiated); BN, binucleated cells; CC, condensed chromatin; KR, karyorrhexis; PY, pyknosis; KL, karyolysis;
