# Supplementary material for: Qualification of the human Reconstructed Intestine Micronuclei Cytome assay for site-of-contact genotoxic hazard identification
Source: NAM J. 2025 Mar 28;1:100015. doi: 10.1016/j.namjnl.2025.100015 (PMC13289030; doi:10.1016/j.namjnl.2025.100015)
Supplement: Supplementary file 3 [file mmc3.pptx]

## Slide 1
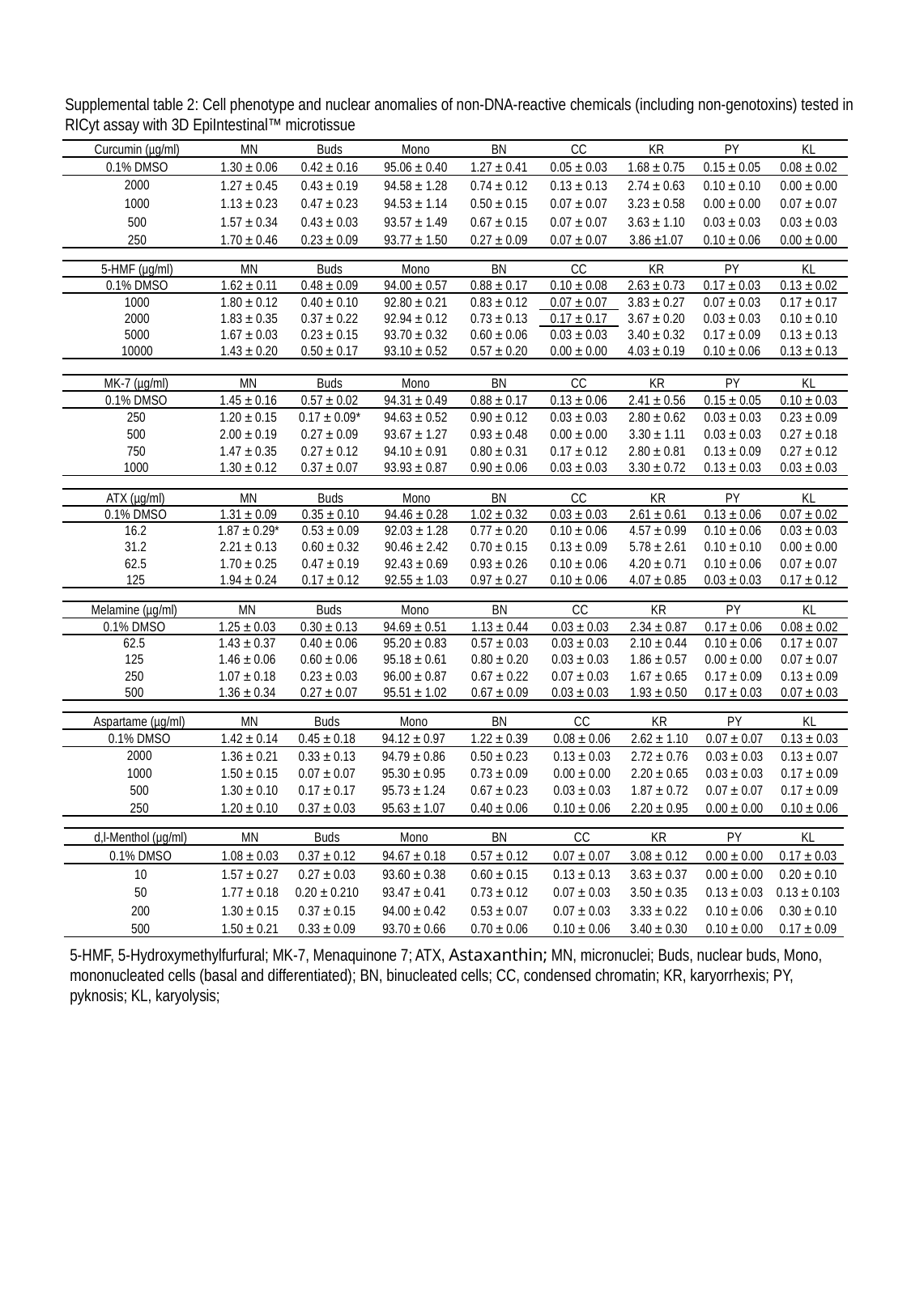

Supplemental table 2: Cell phenotype and nuclear anomalies of non-DNA-reactive chemicals (including non-genotoxins) tested in RICyt assay with 3D EpiIntestinal™ microtissue
| Curcumin (µg/ml) | MN | Buds | Mono | BN | CC | KR | PY | KL |
| --- | --- | --- | --- | --- | --- | --- | --- | --- |
| 0.1% DMSO | 1.30 ± 0.06 | 0.42 ± 0.16 | 95.06 ± 0.40 | 1.27 ± 0.41 | 0.05 ± 0.03 | 1.68 ± 0.75 | 0.15 ± 0.05 | 0.08 ± 0.02 |
| 2000 | 1.27 ± 0.45 | 0.43 ± 0.19 | 94.58 ± 1.28 | 0.74 ± 0.12 | 0.13 ± 0.13 | 2.74 ± 0.63 | 0.10 ± 0.10 | 0.00 ± 0.00 |
| 1000 | 1.13 ± 0.23 | 0.47 ± 0.23 | 94.53 ± 1.14 | 0.50 ± 0.15 | 0.07 ± 0.07 | 3.23 ± 0.58 | 0.00 ± 0.00 | 0.07 ± 0.07 |
| 500 | 1.57 ± 0.34 | 0.43 ± 0.03 | 93.57 ± 1.49 | 0.67 ± 0.15 | 0.07 ± 0.07 | 3.63 ± 1.10 | 0.03 ± 0.03 | 0.03 ± 0.03 |
| 250 | 1.70 ± 0.46 | 0.23 ± 0.09 | 93.77 ± 1.50 | 0.27 ± 0.09 | 0.07 ± 0.07 | 3.86 ±1.07 | 0.10 ± 0.06 | 0.00 ± 0.00 |
| 5-HMF (μg/ml) | MN | Buds | Mono | BN | CC | KR | PY | KL |
| --- | --- | --- | --- | --- | --- | --- | --- | --- |
| 0.1% DMSO | 1.62 ± 0.11 | 0.48 ± 0.09 | 94.00 ± 0.57 | 0.88 ± 0.17 | 0.10 ± 0.08 | 2.63 ± 0.73 | 0.17 ± 0.03 | 0.13 ± 0.02 |
| 1000 | 1.80 ± 0.12 | 0.40 ± 0.10 | 92.80 ± 0.21 | 0.83 ± 0.12 | 0.07 ± 0.07 | 3.83 ± 0.27 | 0.07 ± 0.03 | 0.17 ± 0.17 |
| 2000 | 1.83 ± 0.35 | 0.37 ± 0.22 | 92.94 ± 0.12 | 0.73 ± 0.13 | 0.17 ± 0.17 | 3.67 ± 0.20 | 0.03 ± 0.03 | 0.10 ± 0.10 |
| 5000 | 1.67 ± 0.03 | 0.23 ± 0.15 | 93.70 ± 0.32 | 0.60 ± 0.06 | 0.03 ± 0.03 | 3.40 ± 0.32 | 0.17 ± 0.09 | 0.13 ± 0.13 |
| 10000 | 1.43 ± 0.20 | 0.50 ± 0.17 | 93.10 ± 0.52 | 0.57 ± 0.20 | 0.00 ± 0.00 | 4.03 ± 0.19 | 0.10 ± 0.06 | 0.13 ± 0.13 |
| MK-7 (µg/ml) | MN | Buds | Mono | BN | CC | KR | PY | KL |
| --- | --- | --- | --- | --- | --- | --- | --- | --- |
| 0.1% DMSO | 1.45 ± 0.16 | 0.57 ± 0.02 | 94.31 ± 0.49 | 0.88 ± 0.17 | 0.13 ± 0.06 | 2.41 ± 0.56 | 0.15 ± 0.05 | 0.10 ± 0.03 |
| 250 | 1.20 ± 0.15 | 0.17 ± 0.09\* | 94.63 ± 0.52 | 0.90 ± 0.12 | 0.03 ± 0.03 | 2.80 ± 0.62 | 0.03 ± 0.03 | 0.23 ± 0.09 |
| 500 | 2.00 ± 0.19 | 0.27 ± 0.09 | 93.67 ± 1.27 | 0.93 ± 0.48 | 0.00 ± 0.00 | 3.30 ± 1.11 | 0.03 ± 0.03 | 0.27 ± 0.18 |
| 750 | 1.47 ± 0.35 | 0.27 ± 0.12 | 94.10 ± 0.91 | 0.80 ± 0.31 | 0.17 ± 0.12 | 2.80 ± 0.81 | 0.13 ± 0.09 | 0.27 ± 0.12 |
| 1000 | 1.30 ± 0.12 | 0.37 ± 0.07 | 93.93 ± 0.87 | 0.90 ± 0.06 | 0.03 ± 0.03 | 3.30 ± 0.72 | 0.13 ± 0.03 | 0.03 ± 0.03 |
| ATX (µg/ml) | MN | Buds | Mono | BN | CC | KR | PY | KL |
| --- | --- | --- | --- | --- | --- | --- | --- | --- |
| 0.1% DMSO | 1.31 ± 0.09 | 0.35 ± 0.10 | 94.46 ± 0.28 | 1.02 ± 0.32 | 0.03 ± 0.03 | 2.61 ± 0.61 | 0.13 ± 0.06 | 0.07 ± 0.02 |
| 16.2 | 1.87 ± 0.29\* | 0.53 ± 0.09 | 92.03 ± 1.28 | 0.77 ± 0.20 | 0.10 ± 0.06 | 4.57 ± 0.99 | 0.10 ± 0.06 | 0.03 ± 0.03 |
| 31.2 | 2.21 ± 0.13 | 0.60 ± 0.32 | 90.46 ± 2.42 | 0.70 ± 0.15 | 0.13 ± 0.09 | 5.78 ± 2.61 | 0.10 ± 0.10 | 0.00 ± 0.00 |
| 62.5 | 1.70 ± 0.25 | 0.47 ± 0.19 | 92.43 ± 0.69 | 0.93 ± 0.26 | 0.10 ± 0.06 | 4.20 ± 0.71 | 0.10 ± 0.06 | 0.07 ± 0.07 |
| 125 | 1.94 ± 0.24 | 0.17 ± 0.12 | 92.55 ± 1.03 | 0.97 ± 0.27 | 0.10 ± 0.06 | 4.07 ± 0.85 | 0.03 ± 0.03 | 0.17 ± 0.12 |
| Melamine (µg/ml) | MN | Buds | Mono | BN | CC | KR | PY | KL |
| --- | --- | --- | --- | --- | --- | --- | --- | --- |
| 0.1% DMSO | 1.25 ± 0.03 | 0.30 ± 0.13 | 94.69 ± 0.51 | 1.13 ± 0.44 | 0.03 ± 0.03 | 2.34 ± 0.87 | 0.17 ± 0.06 | 0.08 ± 0.02 |
| 62.5 | 1.43 ± 0.37 | 0.40 ± 0.06 | 95.20 ± 0.83 | 0.57 ± 0.03 | 0.03 ± 0.03 | 2.10 ± 0.44 | 0.10 ± 0.06 | 0.17 ± 0.07 |
| 125 | 1.46 ± 0.06 | 0.60 ± 0.06 | 95.18 ± 0.61 | 0.80 ± 0.20 | 0.03 ± 0.03 | 1.86 ± 0.57 | 0.00 ± 0.00 | 0.07 ± 0.07 |
| 250 | 1.07 ± 0.18 | 0.23 ± 0.03 | 96.00 ± 0.87 | 0.67 ± 0.22 | 0.07 ± 0.03 | 1.67 ± 0.65 | 0.17 ± 0.09 | 0.13 ± 0.09 |
| 500 | 1.36 ± 0.34 | 0.27 ± 0.07 | 95.51 ± 1.02 | 0.67 ± 0.09 | 0.03 ± 0.03 | 1.93 ± 0.50 | 0.17 ± 0.03 | 0.07 ± 0.03 |
| Aspartame (µg/ml) | MN | Buds | Mono | BN | CC | KR | PY | KL |
| --- | --- | --- | --- | --- | --- | --- | --- | --- |
| 0.1% DMSO | 1.42 ± 0.14 | 0.45 ± 0.18 | 94.12 ± 0.97 | 1.22 ± 0.39 | 0.08 ± 0.06 | 2.62 ± 1.10 | 0.07 ± 0.07 | 0.13 ± 0.03 |
| 2000 | 1.36 ± 0.21 | 0.33 ± 0.13 | 94.79 ± 0.86 | 0.50 ± 0.23 | 0.13 ± 0.03 | 2.72 ± 0.76 | 0.03 ± 0.03 | 0.13 ± 0.07 |
| 1000 | 1.50 ± 0.15 | 0.07 ± 0.07 | 95.30 ± 0.95 | 0.73 ± 0.09 | 0.00 ± 0.00 | 2.20 ± 0.65 | 0.03 ± 0.03 | 0.17 ± 0.09 |
| 500 | 1.30 ± 0.10 | 0.17 ± 0.17 | 95.73 ± 1.24 | 0.67 ± 0.23 | 0.03 ± 0.03 | 1.87 ± 0.72 | 0.07 ± 0.07 | 0.17 ± 0.09 |
| 250 | 1.20 ± 0.10 | 0.37 ± 0.03 | 95.63 ± 1.07 | 0.40 ± 0.06 | 0.10 ± 0.06 | 2.20 ± 0.95 | 0.00 ± 0.00 | 0.10 ± 0.06 |
| d,l-Menthol (μg/ml) | MN | Buds | Mono | BN | CC | KR | PY | KL |
| --- | --- | --- | --- | --- | --- | --- | --- | --- |
| 0.1% DMSO | 1.08 ± 0.03 | 0.37 ± 0.12 | 94.67 ± 0.18 | 0.57 ± 0.12 | 0.07 ± 0.07 | 3.08 ± 0.12 | 0.00 ± 0.00 | 0.17 ± 0.03 |
| 10 | 1.57 ± 0.27 | 0.27 ± 0.03 | 93.60 ± 0.38 | 0.60 ± 0.15 | 0.13 ± 0.13 | 3.63 ± 0.37 | 0.00 ± 0.00 | 0.20 ± 0.10 |
| 50 | 1.77 ± 0.18 | 0.20 ± 0.210 | 93.47 ± 0.41 | 0.73 ± 0.12 | 0.07 ± 0.03 | 3.50 ± 0.35 | 0.13 ± 0.03 | 0.13 ± 0.103 |
| 200 | 1.30 ± 0.15 | 0.37 ± 0.15 | 94.00 ± 0.42 | 0.53 ± 0.07 | 0.07 ± 0.03 | 3.33 ± 0.22 | 0.10 ± 0.06 | 0.30 ± 0.10 |
| 500 | 1.50 ± 0.21 | 0.33 ± 0.09 | 93.70 ± 0.66 | 0.70 ± 0.06 | 0.10 ± 0.06 | 3.40 ± 0.30 | 0.10 ± 0.00 | 0.17 ± 0.09 |
5-HMF, 5-Hydroxymethylfurfural; MK-7, Menaquinone 7; ATX, Astaxanthin; MN, micronuclei; Buds, nuclear buds, Mono, mononucleated cells (basal and differentiated); BN, binucleated cells; CC, condensed chromatin; KR, karyorrhexis; PY, pyknosis; KL, karyolysis;
