# Supplementary material for: Qualification of the human Reconstructed Intestine Micronuclei Cytome assay for site-of-contact genotoxic hazard identification
Source: NAM J. 2025 Mar 28;1:100015. doi: 10.1016/j.namjnl.2025.100015 (PMC13289030; doi:10.1016/j.namjnl.2025.100015)
Supplement: Supplementary file 4 [file mmc4.pptx]

## Slide 1
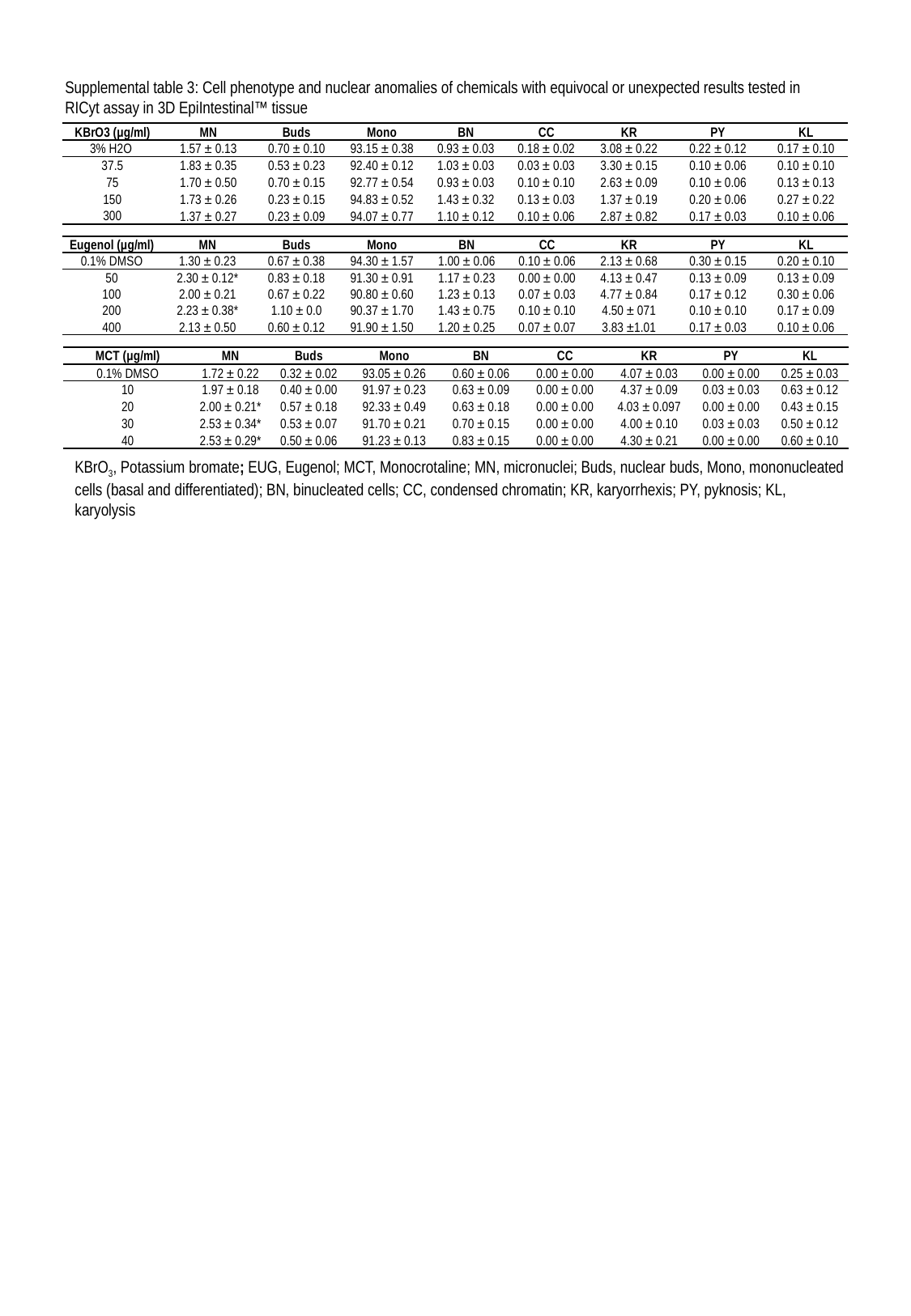

Supplemental table 3: Cell phenotype and nuclear anomalies of chemicals with equivocal or unexpected results tested in RICyt assay in 3D EpiIntestinal™ tissue
| KBrO3 (μg/ml) | MN | Buds | Mono | BN | CC | KR | PY | KL |
| --- | --- | --- | --- | --- | --- | --- | --- | --- |
| 3% H2O | 1.57 ± 0.13 | 0.70 ± 0.10 | 93.15 ± 0.38 | 0.93 ± 0.03 | 0.18 ± 0.02 | 3.08 ± 0.22 | 0.22 ± 0.12 | 0.17 ± 0.10 |
| 37.5 | 1.83 ± 0.35 | 0.53 ± 0.23 | 92.40 ± 0.12 | 1.03 ± 0.03 | 0.03 ± 0.03 | 3.30 ± 0.15 | 0.10 ± 0.06 | 0.10 ± 0.10 |
| 75 | 1.70 ± 0.50 | 0.70 ± 0.15 | 92.77 ± 0.54 | 0.93 ± 0.03 | 0.10 ± 0.10 | 2.63 ± 0.09 | 0.10 ± 0.06 | 0.13 ± 0.13 |
| 150 | 1.73 ± 0.26 | 0.23 ± 0.15 | 94.83 ± 0.52 | 1.43 ± 0.32 | 0.13 ± 0.03 | 1.37 ± 0.19 | 0.20 ± 0.06 | 0.27 ± 0.22 |
| 300 | 1.37 ± 0.27 | 0.23 ± 0.09 | 94.07 ± 0.77 | 1.10 ± 0.12 | 0.10 ± 0.06 | 2.87 ± 0.82 | 0.17 ± 0.03 | 0.10 ± 0.06 |
| Eugenol (μg/ml) | MN | Buds | Mono | BN | CC | KR | PY | KL |
| --- | --- | --- | --- | --- | --- | --- | --- | --- |
| 0.1% DMSO | 1.30 ± 0.23 | 0.67 ± 0.38 | 94.30 ± 1.57 | 1.00 ± 0.06 | 0.10 ± 0.06 | 2.13 ± 0.68 | 0.30 ± 0.15 | 0.20 ± 0.10 |
| 50 | 2.30 ± 0.12\* | 0.83 ± 0.18 | 91.30 ± 0.91 | 1.17 ± 0.23 | 0.00 ± 0.00 | 4.13 ± 0.47 | 0.13 ± 0.09 | 0.13 ± 0.09 |
| 100 | 2.00 ± 0.21 | 0.67 ± 0.22 | 90.80 ± 0.60 | 1.23 ± 0.13 | 0.07 ± 0.03 | 4.77 ± 0.84 | 0.17 ± 0.12 | 0.30 ± 0.06 |
| 200 | 2.23 ± 0.38\* | 1.10 ± 0.0 | 90.37 ± 1.70 | 1.43 ± 0.75 | 0.10 ± 0.10 | 4.50 ± 071 | 0.10 ± 0.10 | 0.17 ± 0.09 |
| 400 | 2.13 ± 0.50 | 0.60 ± 0.12 | 91.90 ± 1.50 | 1.20 ± 0.25 | 0.07 ± 0.07 | 3.83 ±1.01 | 0.17 ± 0.03 | 0.10 ± 0.06 |
| MCT (μg/ml) | MN | Buds | Mono | BN | CC | KR | PY | KL |
| --- | --- | --- | --- | --- | --- | --- | --- | --- |
| 0.1% DMSO | 1.72 ± 0.22 | 0.32 ± 0.02 | 93.05 ± 0.26 | 0.60 ± 0.06 | 0.00 ± 0.00 | 4.07 ± 0.03 | 0.00 ± 0.00 | 0.25 ± 0.03 |
| 10 | 1.97 ± 0.18 | 0.40 ± 0.00 | 91.97 ± 0.23 | 0.63 ± 0.09 | 0.00 ± 0.00 | 4.37 ± 0.09 | 0.03 ± 0.03 | 0.63 ± 0.12 |
| 20 | 2.00 ± 0.21\* | 0.57 ± 0.18 | 92.33 ± 0.49 | 0.63 ± 0.18 | 0.00 ± 0.00 | 4.03 ± 0.097 | 0.00 ± 0.00 | 0.43 ± 0.15 |
| 30 | 2.53 ± 0.34\* | 0.53 ± 0.07 | 91.70 ± 0.21 | 0.70 ± 0.15 | 0.00 ± 0.00 | 4.00 ± 0.10 | 0.03 ± 0.03 | 0.50 ± 0.12 |
| 40 | 2.53 ± 0.29\* | 0.50 ± 0.06 | 91.23 ± 0.13 | 0.83 ± 0.15 | 0.00 ± 0.00 | 4.30 ± 0.21 | 0.00 ± 0.00 | 0.60 ± 0.10 |
KBrO3, Potassium bromate; EUG, Eugenol; MCT, Monocrotaline; MN, micronuclei; Buds, nuclear buds, Mono, mononucleated cells (basal and differentiated); BN, binucleated cells; CC, condensed chromatin; KR, karyorrhexis; PY, pyknosis; KL, karyolysis
